# Synthesis, Characterization, Antibacterial Activity, and Computer-Aided Design of Novel Quinazolin-2,4-dione Derivatives as Potential Inhibitors Against *Vibrio cholerae*

**DOI:** 10.1177/1176934319897596

**Published:** 2020-01-06

**Authors:** Mohamed El-Naggar, Mahmoud Eldeeb Mohamed, Ahmed Mohamed Mosallam, Wesam Salem, Huda RM Rashdan, Aboubakr Haredi Abdelmonsef

**Affiliations:** 1 Chemistry Department, Faculty of Sciences, University of Sharjah, Sharjah, UAE; 2 Chemistry Department, Faculty of Science, South Valley University, Qena, Egypt; 3 Botany and Microbiology Department, Faculty of Science, South Valley University, Qena, Egypt; 4 Chemistry of Natural and Microbial Products Department, Pharmaceutical and Drug Industries Research Division, National Research Centre, Cairo, Egypt

**Keywords:** *Vibrio cholerae*, OmpU, quinazolin-2,4-dione, Gram-negative bacteria, *in silico* docking

## Abstract

Cholera is a bacterial disease featured by dehydration and severe diarrhea. It is mainly caused by alimentary infection with *Vibrio cholerae*. Due to the wide applicability of quinazolin-2,4-dione compounds in medicinal and pharmaceutical chemistry, a new series of *N*-containing heterocyclic compounds was synthesized. We used the *in silico* docking method to test the efficacy of quinazolin-2,4-dione compounds in the prevention of cholera in humans. The newly synthesized compounds showed strong interactions and good binding affinity to outer membrane protein OmpU. Moreover, the pharmacokinetic properties of the newly synthesized compounds, such as absorption, distribution, metabolic, excretion, and toxicity (ADMET), were predicted through *in silico* methods. Compounds with acceptable pharmacokinetic properties were tested as novel ligand molecules. The synthesized compounds were evaluated in vitro for their antibacterial activity properties against Gram-negative *Escherichia coli* O78 strain using the minimum inhibition concentration (MIC) method. Compounds **2** and **6** showed reproducible, effective antibacterial activity. Hence, our study concludes that the quinazolin-2,4-dione derivatives **1** to **8** may be used as promising drug candidates with potential value for the treatment of cholera disease.

## Background

In the last years, the incidence of cholera disease has dramatically increased around the world. Due to its impact, it is considered the world’s most infectious disease, being responsible for 21 000 to 143 000 deaths worldwide. *Vibrio cholerae* is a Gram-negative bacterium that on colonization of the small intestine and induction of virulence factors causes severe diarrhea.^1^ The regulation and expression of its virulence genes have been studied earlier.^2,3^ Like most of the Gram-negative bacteria, *V. cholerae* during their growth produce outer membrane vesicles (OMVs), which entrap periplasm for adherence promotion and transferring bacterial DNA.^4^ A wide variety of *V. cholerae* virulence genes are regulated by *ToxR* and/or *ToxS* that regulates and expresses genes, which encode a porin such as OmpO and OmpT.^5,6^ About 60% of *Vibrio cholera* outer membrane proteins are OmpU if it has grown in salt-free medium.^7^ This same porin forms nonspecific-barrel channels that allow free diffusion of hydrophilic molecules throughout the OM. Apart from its porin function, OmpU has also been shown to confer the pathogen with resistance to bile and antibacterial peptides.^8^ Lembke et al^9^ demonstrated that *ToxR* is blocked independently of *ToxS*; hence, sodium deoxycholate was used for inactivation of *ToxR* by linking bile to *ToxRS* complex formation and further activation of its transcription factor activity. Li et al^10^ reported a high-resolution crystal structure of the outer membrane protein OmpU. Interestingly, the impact of ZnO_2_ nanoparticles on the outer membrane of *Vibrio cholera* affects only the OmpT porins, while OmpU was not affected as recorded by Salem et al.^11^ Consequently, there is a need to identify novel compounds acting as drug-like molecules against cholerae.

In recent years, considerable evidence has been found on the importance of quinolone and tetracycline as having high medicinal value in the treatment of cholera.^12,13^ The nitrogen-containing heterocyclics, such as quinazolindione, are crucial structural motifs of different pharmaceutical compounds and are found in some commercial drugs like ketanserine and pelanseine.^14^ Due to the significant importance of quinazolindione derivatives as anticonvulsant,^14^ antileishmanil,^14^ and antimicrobial agents,^15^ a series of quinazolin-2,4-dione derivatives have been synthesized and *in silico* evaluated to predict the inhibitory activity against the target protein OmpU.^16^ In this context, we have selected OmpU protein as a target for predicting the action of the newly synthesized quinazolin-2,4-dione compounds. However, this study focused on the screening of the newly synthesized compounds against the target protein. PyRx tool is one of such programs which is used to predict the strength of intermolecular docking interactions between the ligand molecules and the binding site of the target protein.^17^ In addition, pharmacokinetics properties of the compounds were also *in silico* predicted.

To extend the applicability of newly quinazolindione derivatives **1** to **8** as antibacterial agents, these newly synthesized compounds were then tested against *Escherichia coli* O78 strain as a model example for the pathogenic Gram-negative bacteria that are very close to *V. cholerae*. Our work aims at offering potential inhibitors, which are novel, cheap, and have less side effects, against *Homo sapiens* OmpU which can be used as drugs for the treatment of cholera disease.

## Results and Discussion

Herein, we reported the synthesis of some new quinazolindione derivatives, and *in silico* molecular docking study was carried out to identify their activity toward cholera disease.

### Chemistry

**Scheme 1.**
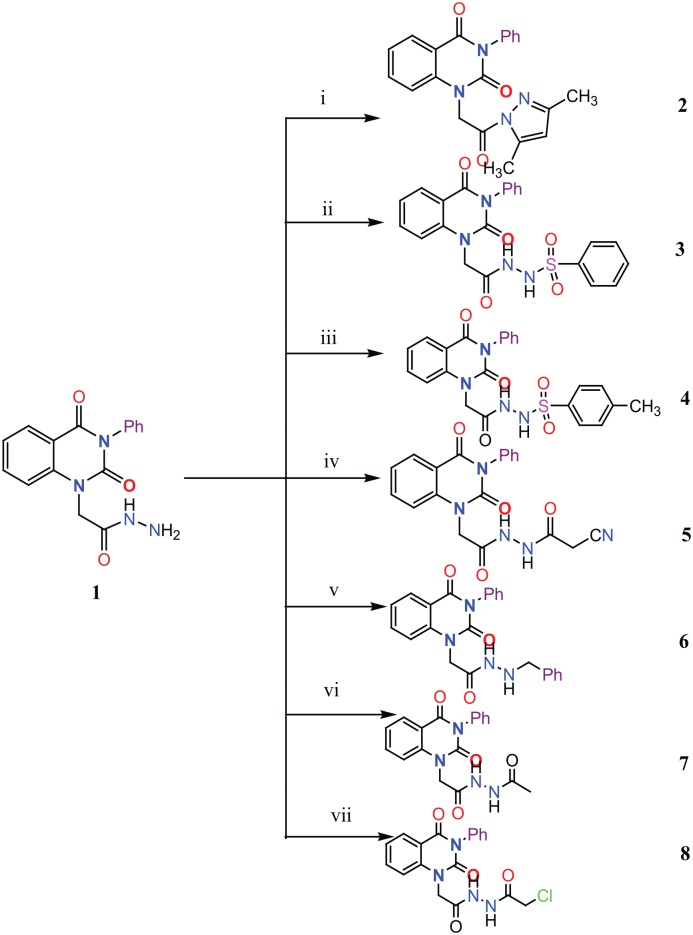
Synthesis of quinazolindione compounds **2** to **8**. (i) Acetylacetone, AcOH, EtOH, and reflux; (ii) benzenesulphonyl chloride, pyridine, and reflux; (iii) toluenesulphonyl chloride, pyridine, and reflux; (iv) ethyl cyanoacetate, EtOH, and reflux; (v) benzyl chloride, EtOH, and reflux; (vi) acetyl chloride, EtOH, and reflux; and (vii) chloroacetyl chloride, EtOH, and reflux.

**Scheme 2.**
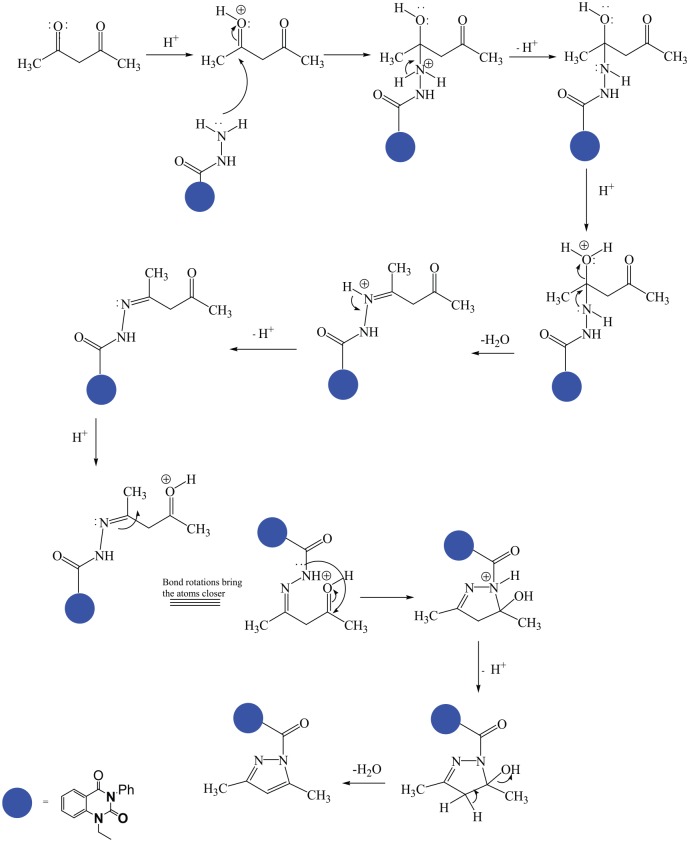
Synthetic strategy of compound **2**.

Eight new quinazolin-2,4-dione derivatives were synthesized by (4-methylene-2-oxo-3-phenyl-3,4-dihydro-2H-quinazolin-1-yl)-acetic acid hydrazide **1** as starting material with different reagents. The title compounds **1** to **8** were synthesized according to Schemes 1 and 2. The spectral data (infrared [IR], ^1^H-NMR, ^13^C-NMR, and mass spectrometry [MS]) of the compounds are represented in Figures S1-S17 (see Supplementary data section). The reaction between *N*-phenylquinazolin-2,4-dione and ethyl chloroacetate in dimethyl formamide (DMF) containing potassium carbonate furnished 2,4-dioxo-3-phenyl-3,4-dihydroquinazolin-1(2H)-yl acetate, which on treatment with hydrazine hydrate in refluxing EtOH afforded the hydrazide derivative **1**. The chemical structure of this hydrazide was established based on its elemental and spectral analyses. The recorded mass at *m/z* 310.31 corresponding to the formula C_16_H_14_N_4_O_3_, beside Infrared spectrum of hydrazide **1**, showed the characteristic absorption bands at wave numbers 3400, 3239, 1687, and 1660 cm^−1^ to indicate the presence of NH_2_, NH, and C=O’s functions, respectively. Furthermore, the strong clue for the structure forthcoming from the 2 singlet signals in the ^1^H-NMR spectrum, each integrated for 2 protons and resonated at δ 3.2 and δ 4.3 ppm, clearly indicated the amino and methylene groups. The aromatic protons resonate as multiplet signals at δ 7.2 to 8.0 ppm. The proton of the NH group was verified as a singlet at δ 11.5 ppm. The synthetic strategy pathway for synthesis of hydrazide **1** is reported earlier in previous works.^18-21^ Then, compound **1** was used as starting material for synthesis of new compounds **2** to **8** as declared in Scheme 1. Furthermore, the new heterocyclic moiety pyrazole in compound **2** was prepared via Knorr pyrazole synthesis by treatment of compound **1** with β-di-ketone such as acetylacetone in 79% yield as reported in Scheme 2.^22^ The compound **2** was identified from spectral and elemental analyses. Thus, the IR spectrum exhibited the characteristic absorption bands at 1664 and 1652 cm^−1^, indicating the presence of functional groups C=Os. The ^1^H-NMR of derivative **2** displayed singlet signals at δ 1.3 and δ 4.3 ppm, indicating the presence of 2 methyl and one methylene groups. The aromatic protons resonated as multiplet signals at δ 7.10 to 8.07 ppm. Also, when compound **1** was treated with benzene and/or toluene, sulphonylchloride afforded benzene sulphonyl N′-[2-(4-Methylene-2-oxo-3-phenyl-3,4-dihydro-2H-quinazolin-1-yl)-acetyl]-hydrazide **3** and Toluenesulphonyl N′-[2-(4-Methylene-2-oxo-3-phenyl-3,4-dihydro-2H-quinazolin-1-yl)-acetyl]-hydrazide **4**. Similar spectral analyses were conducted for derivatives **3** and **4**. Infrared spectrum exhibited the same characteristic absorption bands at close ranges for the functional groups NH and C=O’s groups. The ^1^H-NMR spectrum of derivatives **3** and **4** is similar to each other. Extra peak as singlet signals at δ 1.3 ppm indicates the presence of methyl group in compound **4**. The starting material **1** reacted with an equimolar amount of ethyl cyanoacetate to yield compound **5**. The IR spectrum of the isolated product confirmed the presence of an intense absorption band centered around 2270 cm^−1^ because of (C≡N) cyano group. Its mass spectrum declared the molecular ion at *m/z* 377 corresponding to its molecular formula C_19_H_15_N_5_O_4_. Furthermore, the reaction of hydrazide **1** with benzyl chloride was achieved to afford the corresponding (2,4-dioxo-3-phenyl-3,4-dihydro-2*H*-quinazolin-1-yl)-acetic acid N′-benzyl-hydrazide **6**. Structure proof of compound **6** was deduced from its spectral analysis, where its mass spectrum recorded molecular ion peak (C_23_H_20_N_4_O_3_) at *m/z* 400, while the IR spectrum showed characteristic absorption bands at 3415 and 1652 to 1670 cm^−1^ because of the presence of (NH) and C=Os, respectively. However, treatment of key compound **1** with acetyl chloride and chloroacetyl chloride afforded acetic acid N′-[2-(2,4-dioxo-3-phenyl-3,4-dihydro-2H-quinazolin-1-yl)-acetyl]-hydrazide **7** and chloro acetic acid N′-[2-(2,4-dioxo-3-phenyl-3,4-dihydro-2H-quinazolin-1-yl)-acetyl]-hydrazide **8**. The structural confirmations of these compounds were proved by elemental analysis and spectral data as declared in the “Experimental” section.

### In silico study

**Figure 1.**
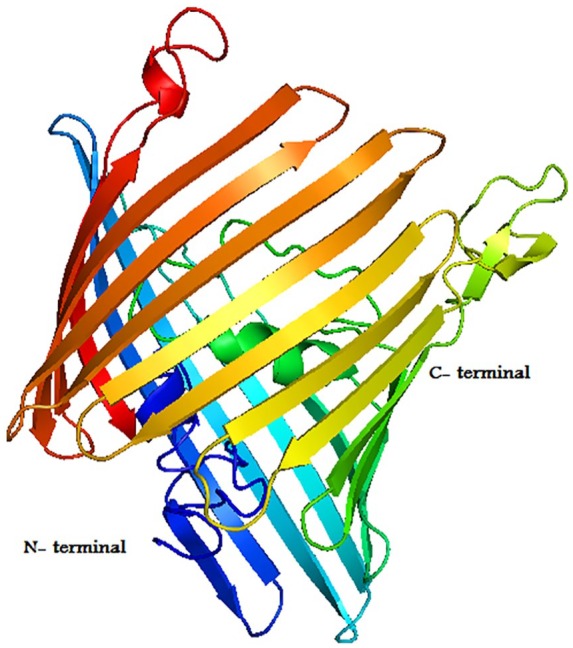
The three-dimensional (3D) structure of OmpU protein. The 3D model of OmpU consists of 6 α-helices and 21 β-strands as predicted from PDBsum. N-terminal indicates the starting residue and C-terminal indicates the end residue.

The outer membrane protein OmpU is selected as a target protein for the identification of novel leads against cholera disease. Recently, the crystal structure of the outer membrane protein OmpU (PDBID: 5ONU) of 341 amino acid residues, with a high resolution, was reported by Li et al, which is a significant virulence factor in *V cholerae* and delivers a vigorous mechanical basis for the colonization of host cells surfaces and the phage-recognition mechanism. The crystallographic structure of OmpU was downloaded from protein data bank website (rcsb.org) as shown in Figure 1.

**Figure 2.**
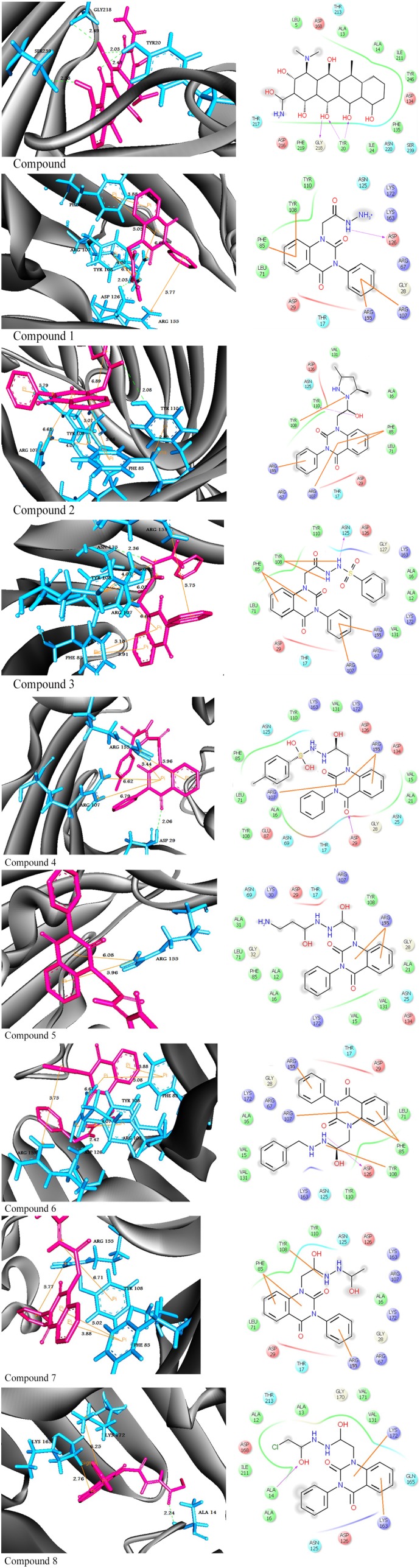
Molecular interactions of compounds **1** to **8** and reference with OmpU protein. (Left side) Three-dimensional representation: the amino acid residues in the binding cavity of OmpU are represented in cyan stick models and the compounds are in pink stick models. The hydrogen bond is represented in green dotted lines. (Right side) Two-dimensional representation: the amino acids are shown in 3-letter code and H-bonds in pink lines. π-interactions are shown in yellow lines.

Furthermore, the physicochemical properties of the target protein were predicted using the ProtParam tool.^23^ The molecular weight was 36 645 Da and the isoelectric point (pI) was 4.46. The results show that the amino acid residues ALA, GLY, ASP, ASN, TYR, and VAL were present in high percentages in OmpU protein. In molecular docking method, all the synthesized compounds were docked into the binding site of OmpU. The binding site prediction tools such as MetaPocket and ProBiS declare that the amino acid residues ALA14, ASP29, ARG107-TYR110, ASN125-ASP126, and ARG155-LYS172 are crucial for OmpU to bind with quinazolindione derivatives. An in-house database of 8 quinazolindione compounds was created in standard file format (SDF). All the compounds were energy minimized using Universal Force Field (UFF),^24^ to obtain structures with proper bond length between different atoms, and then used in the virtual screening study. In addition, doxycycline was chosen as a reference compound to compare the docking score of synthesized compounds. Herein, PyRx software was employed to simulate the ligand into the active site of the protein to calculate the binding energy of the ligand-receptor complexes. Computer-based docking predicted 9 conformers for each ligand-protein complex. The preferable binding orientation between protein and compound, with more negative binding affinity score, was considered for further study.^25^ Docking interactions between compounds and protein are shown in Figure 2. The docked ligand molecules scored good binding affinity values of −10.6 to −8.0 kcal/mol. Compound **1** interacts with protein at ASP126, ARG107, TYR108, and ARG155 forming one H-bond (O–H–O) and 3 pi-cation interactions. Compound **2** exhibited H-bond (N–H–O) and pi-cation interactions with TYR110, ARG107, TYR108, and ARG155, respectively. In addition, compound **3** formed H-bond with ASN125 (N–H–O) and pi-cation interactions with ARG107, TYR108, and ARG155. While compounds **4** and **6** formed H-bonds with ASP29 (N–H–O) and ASP126 (O–H–N) and pi-cation interactions with ARG107, TYR108, and ARG155. The derivate **5** exhibited pi-cation interactions with ARG155. The derivative **7** exhibited pi-cation interactions with TYR108 and ARG155. Finally, compound **8** showed H-bond interaction with ALA14 (O–H–N), pi-cation interaction with LYS172, and pi-sigma interaction with LYS163. All the synthesized compounds except **4**, **5**, and **8** exhibited pi-pi interactions with PHE85. The results showed that all compounds **1** to **8** form pi-cation interactions with the target proteins through ARG107, LYS108, and ARG155. This is due to arginine containing positively charged guanidinium group H_2_N–C(=NH)–NH– that is involved in forming pi-cation interaction with pyrimidine moiety in compounds **1** to **7**. However, lysine contains a positively charged amino on its side chain that is involved in forming pi-cation interaction with pyrimidine moiety of compound **8**. Clearly, pyrimidine and amide moieties are observed to be common pharmacophore groups, which interact with the binding residues of OmpU protein through various interactions as represented in Table 1. However, doxycycline which is used as a reference has the binding affinity (−10.7 kcal/mol) closer to the synthesized compounds and exhibited 4 H-bond interactions with TYR20, GLY219, and SER239, respectively. It was observed that all the compounds showed strong interactions to OmpU through hydrogen and non-covalent bonds such as pi-interactions. Lower binding energy and more non-covalent bonds imply best binding stability and inhibitory characteristic^26,27^ of the synthesized compounds against cholera.

**Table 1.** The binding affinity (kcal/mol) of various compounds **1** to **8** and reference with OmpU after molecular docking.

	Structure	Binding affinity (kcal/mol)	Docked complex (amino acid -ligand) interactions	Distance (Å)
Ref	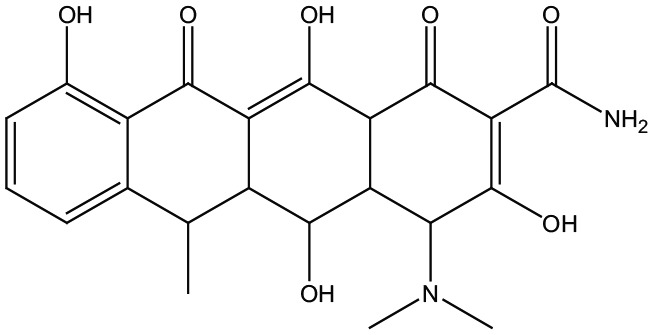	−10.7	H-bonds	
			Reference—TYR20: HH	2.03
			Reference—TYR20: HH	2.48
			Reference—SER239: HG	2.38
			Reference—GLY218: O	2.49
1	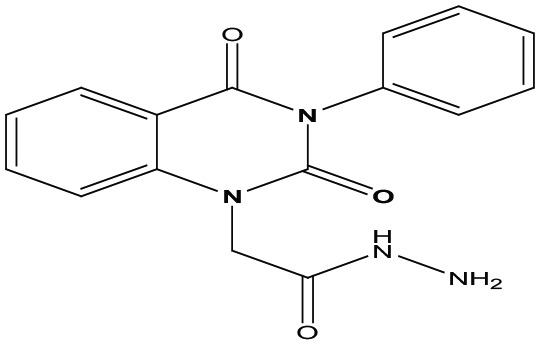	−8.0	H-bonds	
			Compound 1—ASP126: OD2	2.03
			π-π interactions	
			Compound 1—PHE85	3.88
			Compound 1—PHE85	5.05
			π-cation interactions	
			Compound 1—ARG107: NH1	4.07
			Compound 1—ARG155: NH1	5.77
			Compound 1—TYR108	6.17
2	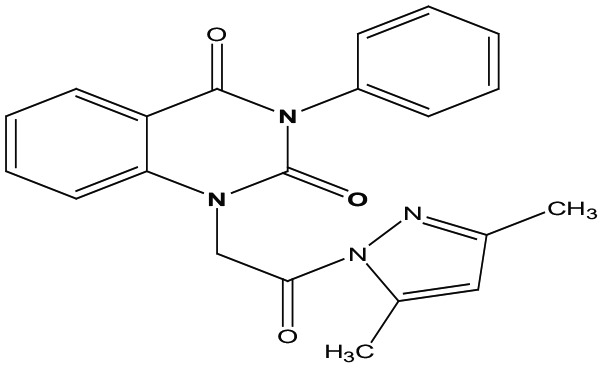	−9.6	H-bonds	
			Compound 2—TYR110:OH	2.08
			π-π interactions	
			Compound 2—PHE85	3.86
			Compound 2—PHE85	5.07
			π-cation interactions	
			Compound 2—ARG107: NH1	6.63
			Compound 2—ARG155: NH1	5.79
			Compound 2—TYR108	4.07
3	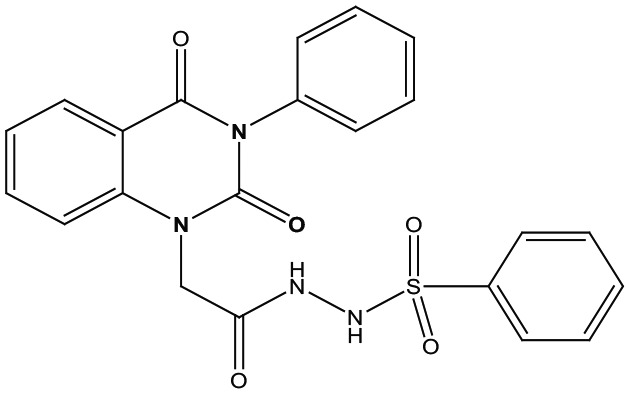	−10.6	H-bonds	
			Compound 3—ASN125:OD1	2.36
			π-π interactions	
			Compound 3—PHE85	3.91
			Compound 3—PHE85	5.10
			π-cation interactions	
			Compound 3—ARG107:NH1	6.64
			Compound 3—ARG155:NH1	5.75
			Compound 3—TYR108	4.07
			Compound 3—TYR108	6.02
4	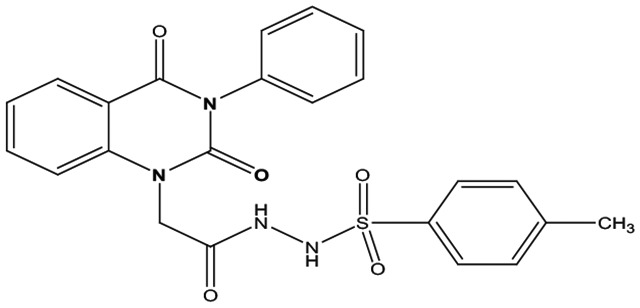	−10.0	H-bonds	
			Compound 4—ASP29:H	2.06
			π-cation interactions	
			Compound 4—ARG107:NH1	6.62
			Compound 4—ARG107:NH1	6.79
			Compound 4—ARG155:NH1	5.44
			Compound 4—ARG155:NH1	5.96
5	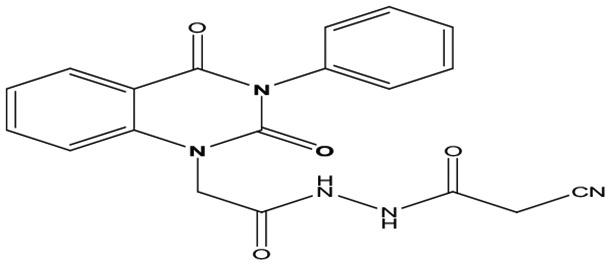	−9.0	π-cation interactions	
			Compound 5—ARG155:NH1	5.96
			Compound 5—ARG155:NH1	6.08
6	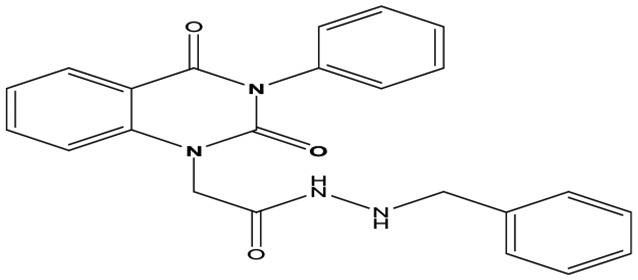	−9.5	H-bonds	
			Compound 6—ASP126:OD2	2.42
			π-π interactions	
			Compound 6—PHE85	3.88
			Compound 6—PHE85	5.08
			π-cation interactions	
			Compound 6—ARG107:NH1	6.67
			Compound 6—ARG155:NH1	5.75
			Compound 6—TYR108	4.07
7	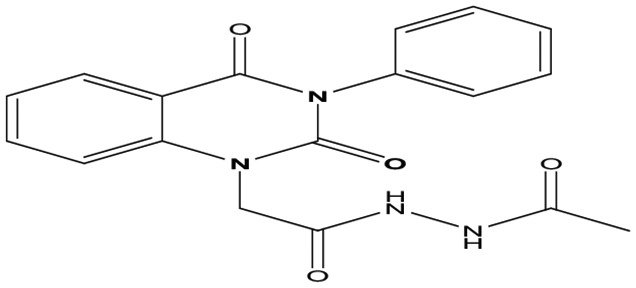	−8.8	π-π interactions	
			Compound 7—PHE85	3.88
			Compound 7—PHE85	5.02
			π-cation interactions	
			Compound 7—ARG155:NH1	5.77
			Compound 7—TYR108	6.71
8	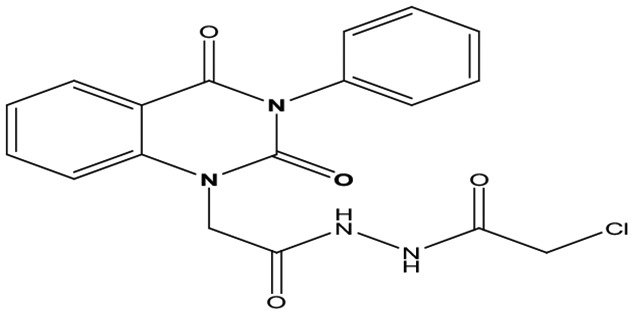	−8.7	H-bonds	
			Compound 8—ALA14:H	2.24
			π-cation interactions	
			Compound 8—LYS172:NZ	6.25
			π-sigma interactions	
			Compound 8—LYS163:HB2	2.76

The 8 molecules (**1-8**) with the best binding affinity are represented with docking interactions in the table showing H-bonding, pi-pi, pi-cation, and pi-sigma interactions.

Drug-likeness scores of the screened quinazolindione compounds were filtered by *in silico* prediction of ADMET, using admetSAR.^28^ The compounds with acceptable ADMET properties in the permissible range are considered as novel molecules.^29^ The pharmacological properties of 8 compounds **1** to **8** are presented in Table 2. Interestingly, all the newly synthesized compounds have a molecular weight in the range of 310 to 464 (<500). Blood-brain barrier (BBB)^+^ value describes the ability of the compounds to cross the BBB, which is in the permissible ranges for all compounds. Also, the values show that the compounds can be absorbed by the human intestines and noncarcinogenic. The results show that these compounds show better inhibition properties against OmpU protein and promising pharmacokinetics properties.

**Table 2.** List of ADMET properties of the newly synthesized molecules (**1-8**).

	Molecular weight (g/mol)	Blood-brain barrier (BBB+)	Human intestinal absorption (HIA+)	Caco-2 permeability (Caco-2+)	Ames toxicity	Carcinogenicity
1	310	0.96	1.00	0.58	Nontoxic	Noncarcinogenic
2	376	0.83	0.99	0.64	Nontoxic	Noncarcinogenic
3	450	0.61	0.95	0.66	Nontoxic	Noncarcinogenic
4	464	0.53	0.97	0.65	Nontoxic	Noncarcinogenic
5	377	0.89	0.99	0.61	Nontoxic	Noncarcinogenic
6	400	0.94	1.00	0.60	Nontoxic	Noncarcinogenic
7	352	0.85	0.99	0.61	Nontoxic	Noncarcinogenic
8	386	0.87	1.00	0.61	Nontoxic	Noncarcinogenic

Abbreviation: ADMET, absorption, distribution, metabolic, excretion, and toxicity.

The pharmacokinetic properties of the molecules (**1-8**) which form docked complexes with OmpU protein are evaluated by admetSAR. The agreeable ranges are as follows: mol. wt.: 130 to 725; %human oral absorption: >80% high and <25% low.

Our study concluded that the newly synthesized quinazolin-2,4-dione compounds such as (4-methylene-2-oxo-3-phenyl-3,4-dihydro-2H-quinazolin-1-yl)-acetic acid hydrazide **1**, 1-[2-(3,5-dimethyl-4,5-dihydro-pyrazol-1-yl)-2-oxo-ethyl]-3-phenyl-1*H*-quinazolin-2,4-dione **2**, benzenesulphonyl N′-[2-(4-methylene-2-oxo-3-phenyl-3,4-dihydro-2*H*-quinazolin-1-yl]-acetyl]-hydrazide **3**, toluenesulphonyl N′-[2-(4-methylene-2-oxo-3-phenyl-3,4-dihydro-2*H*-quinazolin-1-yl)-acetyl]-hydrazide **4**, cyano acetic acid N′-[2-(2,4-dioxo-3-phenyl-3,4-dihydro-2*H*-quinazolin-1-yl)-acetyl]-hydrazide **5**, (2,4-dioxo-3-phenyl-3,4-dihydro-2*H*-quinazolin-1-yl)-acetic acid N′-benzyl-hydrazide **6**, acetic acid N′-[2-(2,4-dioxo-3-phenyl-3,4-dihydro-2*H*-quinazolin-1-yl)-acetyl]-hydrazide **7**, and chloroacetic acid N′-[2-(2,4-dioxo-3-phenyl-3,4-dihydro-2*H*-quinazolin-1-yl)-acetyl] hydrazide **8** are of great importance and good inhibitors, which can be used as potential drug-like molecules against cholera. Hence, the OmpU function as pore selectivity helps *V cholerae* for persistence within diverse extracellular environments, which consider a challenge faced by a respective host (human). The distinct quinazolindione structural features in this study create an informative platform for blocking the function and biophysical properties of OmpU.

### Antibacterial activity of quinazolindione compounds against E. coli O78 strain

**Figure 3.**
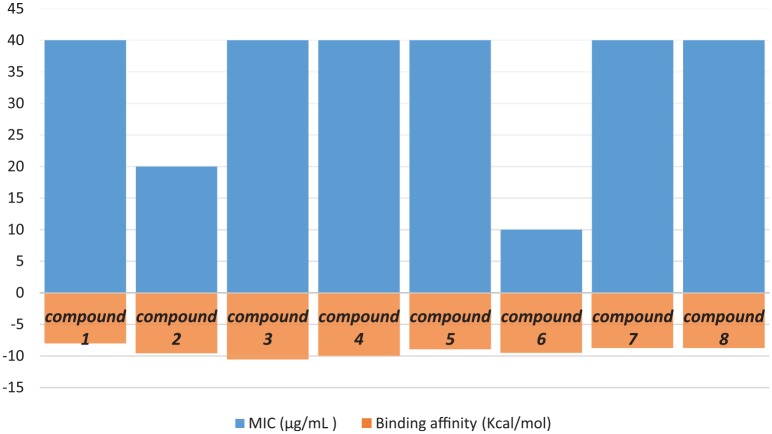
The relationship between the antibacterial activity and binding affinity of all synthesized compounds **1** to **8**.

Eight newly synthesized compounds **1** to **8** were screened for their *in vitro* antibacterial activities against Gram-negative strain (*E. coli* O78 strain) by using the minimum inhibitory concentration (MIC) method. The relationship between the reactivity and binding affinity of all synthesized compounds against bacterial specie was evaluated and summarized as MIC values in µg/mL in Figure 3 and Table 3.

**Table 3.** Minimum inhibitory concentration (MIC) values of the quinazolindione derivatives against Gram-negative *Escherichia coli* O78 strain.

Compound no.	MIC (µg/mL) after 24 hours
1	40
2	20
3	40
4	40
5	40
6	10
7	40
8	40

The compounds **2** and **6** exhibited the highest effect against Gram-negative *E. coli* O78 strain.

### The MICs

The colorimetric iodonitrotetrazolium (INT)-formazon assay of compounds **2** and **6** showed reproducible, efficacious antibacterial activity with MICs values of 20 and 10 µg/mL, respectively, against the tested *E. coli* strain as represented in Figure S18 in the Supplementary data section. The result implicated that the presence of pyrazole moiety and benzyl group is essential for the antibacterial activity of these compounds.^30,31^

## Methods

### Chemistry

#### Materials and apparatus

All melting points of the compounds were determined on Griffin apparatus and are uncorrected. The IR spectra of samples were recorded in KBr via a Shimadzu FT-IR 8101 PC infrared spectrophotometer. The NMR spectra were run at 400 MHz using tetramethylsilane (TMS) as the internal standard. Chemical shifts were measured in ppm (δ) related to TMS (0.00 ppm). Mass spectra were measured on a GCMS-QP1000 EX spectrometer at 70 eV. Elemental analyses were carried out at the Micro Analytical Center of Cairo University, Giza, Egypt. Thin-layer chromatography (TLC) was conducted on 0.25 mm precoated silica gel plates (60F-254). Purities of these compounds were established by TLC.

#### Spectral analysis of quinazoline compounds

##### (4-Methylene-2-oxo-3-phenyl-3,4-dihydro-2H-quinazolin-1-yl)-acetic acid hydrazide **1**

A mixture of (2,4-dioxo-3-phenyl-3,4-dihydro-2H-quinazolin-1-yl)-acetic acid ethyl ester (1 mmol) and hydrazine hydrate (1 mmol) in absolute ethanol was refluxed for 6 hours; after cooling, the solid formed was filtered off and recrystallized from ethanol to give compound **1** as white crystal; yield 76%; m.p. 210 to 212. Fourier-transform infrared (FT-IR; KBr, υ, cm^−1^) = 3400, 3239 (N–H stretch of NH_2_ and NH), 1660, and 1687 (C=O). ^1^H-NMR (DMSO d6, 400 MHz): δ (ppm) = 11.5 (s, 1H, NH), 7.2 to 8.0 (m, 9H, Ar-H), 8.8 (s, 2H, NH_2_), and 4.3 (s, 2H, CH_2_).^13^C-NMR (CDCl_3_): δ 49.8, 114.4, 115.3, 124.7, 125.1, 126.5, 128.6, 128.8, 129.3, 129.5, 131.8, 136.2, 140.7, 151.9, 161.3, 165.8. MS (electron ionization [EI]): *m/z* (%) = 310.31 [M]^+^. *Anal. calcd* for C_16_H_14_N_4_O_3_: C, 61.93%; H, 4.55%; and N, 18.05%. Found C, 61.96%; H, 4.57%; N, 18.11%.

##### 1-[2-(3,5-Dimethyl-4,5-dihydro-pyrazol-1-yl)-2-oxo-ethyl]-3-phenyl-1H-quinazolin-2,4-dione **2**

To a solution of compound **1** (1 mmol) in ethanol, acetylacetone (1 mmol) was added in the presence of catalytic drops of glacial acetic acid, and the reaction mixture was refluxed for 8 hours; after cooling, a solid product was filtered off and recrystallized from ethanol to afford compound **2** as light yellow powder; yield 82%; m.p. 255 to 257. FT-IR (KBr, υ, cm^−1^) = (C=O): 1652 and 1664. ^1^H-NMR (DMSO d6, 400 MHz): δ (ppm) = 7.2 to 8.0 (m, 10H, Ar-H&CH), 4.3 (s, 2H, CH_2_), and 1.3 (s, 6H, 2CH_3_).^13^C-NMR (CDCl_3_): δ 12.7, 13.4, 49.8, 110.5,114.4, 115.3, 124.7, 125.1, 126.5, 128.6, 128.8, 129.1, 129.3, 131.8, 136.2, 139.5, 140.7, 149.0, 151.9, 161.3, 167.3. MS (EI): *m/z* (%) = 374 [M]^+^. *Anal. calcd* for C_21_H_18_N_4_O_3_: C, 67.37%; H, 4.85%; N, 14.96%. Found C, 67.42%; H, 4.87%; N, 14.92%.

##### Arylsulfonyl N′-[2-(4-methylene-2-oxo-3-phenyl-3,4-dihydro-2H-quinazolin-1-yl)-acetyl]-hydrazide **3-4**

A stirred solution of 4-methylene-2-oxo-3-phenyl-3,4-dihydro-2H-quinazolin-1-yl-acetic acid hydrazide **1** (1 mmol) in dry pyridine (10 mL) was cooled in an ice bath; benzene and/or toluenesulphonyl chloride(1 mmol) was added dropwise. The reaction mixture was stirred at room temperature for 4 hours. A cold diluted HCl (1:1) was added to the reaction mixture, and the solid formed was filtered off and recrystallized from ethanol/benzene to give compounds **3** and **4**, respectively, as white powders.

##### Benzenesulphonyl N′-[2-(4-Methylene-2-oxo-3-phenyl-3,4-dihydro-2H-quinazolin-1-yl)-acetyl]-hydrazide **3**

Yield 74%; m.p. 240 to 241. FT-IR (KBr, υ, cm^−1^) = (NH), 3380 (C=Os), 1663, and 1685. ^1^H-NMR (DMSO d6, 400 MHz): δ (ppm) = 11.5 (s, 1H, NH), 7.2 to 8.0 (m, 14H, Ar-H), and 4.3 (s, 2H, CH_2_). MS (EI): *m/z* (%) = 450 [M]^+^. *Anal. calcd* for C_22_H_18_N_4_O_5_S: C, 58.66%; H, 4.03%; N, 12.44%. Found C, 58.90%; H, 4.32%; N, 12.71%.

##### Toluenesulphonyl N′-[2-(4-Methylene-2-oxo-3-phenyl-3,4-dihydro-2H-quinazolin-1-yl)-acetyl]-hydrazide **4**

Yield 78%; m.p. 270 to 272. FT-IR (KBr, υ, cm^−1^) = (NH), 3365 (C=Os), 1660, and 1690. ^1^H-NMR (DMSO d6, 400 MHz): δ (ppm) = 11.5 (s, 2H, 2NH), 7.2 to 8.0 (m, 13H, Ar-H), 4.3 (s, 2H, CH_2_), and 1.3 (s, 3H, CH_3_). MS (EI): *m/z* (%) = 464 [M]^+^. *Anal. calcd* for C_23_H_20_N_4_O_5_S: C, 9.47%; H, 4.34%; N, 12.06%. Found C, 59.50%; H, 4.65%; N, 12.15%.

##### Cyano-acetic acid N′-[2-(2,4-dioxo-3-phenyl-3,4-dihydro-2H-quinazolin-1-yl)-acetyl]-hydrazide **5**

Heating of hydrazide **1** (1 mmol) with ethyl cyanoacetate (1 mmol) in ethanol (20 mL) under reflux for 8 hours gave after cooling a solid product, which was filtered off and recrystallized from ethanol/benzene to give compound **5** as yellow crystal; yield 81%; m.p. 260 to 262. FT-IR (KBr, υ, cm^−1^) = (NH), 3477 (C = Os), 1667 and 1688 (CN), and 2209. ^1^H-NMR (DMSO d6, 400 MHz): δ (ppm) = 11.5 (s, 1H, NH), 7.5 to 8.0 (m, 9H, Ar-H), 5.6 (s, 2H, CH_2_–C=O), 4.3 (s, 2H, CH_2_–CN). MS (EI): *m/z* (%) = 377 [M]^+^. *Anal. calcd* for C_19_H_15_N_5_O_4_: C, 60.48%; H, 4.01%; N, 18.56%. Found C, 60.65%; H, 4.19%; N, 18.72%.

##### (2,4-Dioxo-3-phenyl-3,4-dihydro-2H-quinazolin-1-yl)-acetic acid N′-benzyl-hydrazide **6**

A mixture of (4-methylene-2-oxo-3-phenyl-3,4-dihydro-2*H*-quinazolin-1-yl)-acetic acid hydrazide **1** (1 mmol) and benzyl chloride (1 mmol) in 20 mL ethanol was refluxed for 7 hours. After cooling, a solid product was filtered off and recrystallized from ethanol/benzene to give compound **6** as gray crystals; yield 72%; m.p. 290 to 292. FT-IR (KBr, υ, cm^−1^) = (NH), 3415 (C=Os), 1652, and 1670. ^1^H-NMR (DMSO d6, 400 MHz): δ (ppm) = 11.5 (s, 1H, NH), 7.2 to 8.0 (m, 14H, arom.), 4.3 (s, 2H, CH_2_-Ph), and 3.9 (s, 2H, CH_2_–CO).^13^C-NMR (CDCl_3_): δ49.3, 49.8,114.4, 115.3,124.7,125.1, 126.5, 127.5, 127.7, 128.2, 128.4, 128.6, 128.8, 128.9, 129.1, 129.3, 131.8, 136.2, 137.5, 140.7, 151.9, 161.3, 165.8. MS (EI): *m/z* (%) = 400 [M]^+^. *Anal. calcd* for C_23_H_20_N_4_O_3_: C, 68.99%; H, 5.03%; N, 13.99%. Found C, 67.11%; H, 5.17%; N, 14.22%.

##### Acetic acid N′-[2-(2,4-dioxo-3-phenyl-3,4-dihydro-2H-quinazolin-1-yl)-acetyl]-hydrazide **7**

(4-Methylene-2-oxo-3-phenyl-3,4-dihydro-2*H*-quinazolin-1-yl)-acetic acid hydrazide **1** (1 mmol) was heated under reflux for 8 hours with acetyl chloride (1 mmol) in absolute ethanol (20 mL). After cooling, a solid product was filtered off and recrystallized from ethanol/benzene to afford compound **7** as pale yellow crystals; yield 85%; m.p. 244 to 246. FT-IR (KBr, υ, cm^−1^) = (NH), 3428 (C=Os) 1648, and 1674. ^1^H-NMR (DMSO d6, 400 MHz): δ (ppm) = 11.5 (s, 2H, 2 NH), 7.7 to 8.0 (m, 9H, Ar-H), 4.3 (s, 2H, CH_2_), 2.3 (s, 3H, CH_3_).^13^C-NMR (CDCl_3_): δ20.5, 49.8, 114.4, 115.3, 124.7, 125.1, 126.5, 128.6, 128.8, 129.3, 129.5, 131.8, 136.2, 140.7, 151.9, 161.3, 165.8, 167.9. MS (EI): *m/z* (%) = 352 [M]^+^. *Anal. calcd* for C_18_H_16_N_4_O_4_: C, 61.36%; H, 4.58%; N, 15.90%. Found C, 61.55%; H, 4.72%; N, 16.23%.

##### Chloro-acetic acid N′-[2-(2,4-dioxo-3-phenyl-3,4-dihydro-2H-quinazolin-1-yl)-acetyl]-hydrazide **8**

To a solution of (4-methylene-2-oxo-3-phenyl-3,4-dihydro-2*H*-quinazolin-1-yl)-acetic acid hydrazide **1** (1 mmol) in ethanol, chloroacetyl chloride (1 mmol) was added, and the reaction mixture was refluxed for 6 hours. After cooling, a solid product was filtered off and recrystallized from ethanol/benzene to give compound **8** as white crystals; yield 86%; m.p. 270 to 271. FT-IR (KBr, υ, cm^−1^) = (NH), 3416 (C=Os), 1643, and 1665. ^1^H-NMR (DMSO d6, 400 MHz): δ (ppm) = 11.5 (s, 1H, NH), 8.7 (s, 1H, NH), 7.2 to 8.0 (m, 9H, Ar-H), 4.3 (s, 2H, CH_2_), 4.2 (s, 2H, CH_2_).^13^C-NMR (CDCl_3_): δ 42.6, 49.8, 114.4, 115.3, 124.7, 125.1, 126.5, 128.8, 129.3, 129.5, 130.0, 131.8, 136.2, 140.7, 151.9, 161.3, 165.8, 167.3. MS (EI): *m/z* (%) = 386.80 [M]^+^ and 388.80 [M^+^ + 2] due to the presence of chlorine atom. *Anal. calcd* for C_18_H_15_N_4_O_4_Cl: C, 55.90%; H, 3.91%; N, 14.48%. Found C, 56.13%; H, 4.05%; N, 14.72%.

## *In Silico* Study

### Binding site identification

Various computational methods were used for the prediction of drug-like molecules against cholera. The binding site region was identified using computational prediction tools like MetaPocket2.0 and ProBiS.^32,33^ A grid with dimensions 25A° × 25A° × 25A° is created around the binding site of OmpU to perform the screening studies.

### In silico docking and ADME prediction

Next, *in silico* molecular docking of 8 quinazolindione derivatives and reference compound onto the target has been carried out using PyRx-virtual screening tool. The intermolecular interactions between the docked compounds-protein complexes such as H-bonds, π-π, π-cation, and π-sigma interactions add more stability to the target protein.^34^ Because of these interactions, compounds are arranged in a certain position within a protein. Discovery Studio^®^ Visualizer 2016 software (Accelrys Software Inc., San Diego, CA, USA) was used to visualize the interactions. In addition, the prediction of absorption, distribution, metabolic, excretion, and toxicity (ADMET) properties plays a key role in drug design. Blood-brain barrier, human intestinal absorption (HIA), Caco-2 Permeability (Caco2+), Ames toxicity and carcinogenicity were calculated using admetSAR (http://lmmd.ecust.edu.cn/admetsar1/). The promising results for the tested compounds indicate that they can be used as drug candidates.

### Biological screening (antibacterial activity)

The antibacterial activity of compounds **1** to **8** was estimated by the minimum inhibition concentration (MIC) method. *Escherichia coli* O78 is the test strain selection pathogenic bacteria in our work. *Escherichia coli* was kindly donated from the bacteriology Lab.- Faculty of Science, South Valley University. An amount of 0.2 g of each compound was dissolved in 1 mL dimethyl sulfoxide (DMSO) and completed with sterile tryptic soy broth (TSB) medium to 10 mL to avoid the inhibitory effect of DMSO (10-fold dilution).^35^ Overnight culture of *E. coli* was diluted to 1:10.000 into TSB. Bacterial growth was placed into 96-well plates plus different amounts of quinazolindione derivatives. After 24 hours of incubation at 37°C, the MIC was the lowest concentration that inhibited bacterial growth. To confirm bacterial growth inhibition and lack of metabolic activity, 40 µL of *p*-iodo nitrotetrazolium violet (INT) (0.2 µg/mL; Sigma-Aldrich) was added to the microplate wells and re-incubated at 37°C for 30 minutes.^36^ The MIC in the INT assay was defined as the lowest concentration that prevented color change as described earlier by Lall et al.^37^

## Conclusions

Cholera is common in places with famine and poor sanitation that can cause acute diarrhea. This study provides an insight into the efficiency of quinazolindione derivatives as new chemical entities (NCEs) against cholera. The resulted data from *in silico* study are helpful to identify new potential lead molecules for the treatment of cholera throughout the blocking mechanisms of OmpU binding sites. The antibacterial activities of the newly synthesized compounds were evaluated and revealed that the compounds **2** and **6** are the most effective against Gram-negative bacterial strains.

## Supplemental Material

Supplementary__File – Supplemental material for Synthesis, Characterization, Antibacterial Activity, and Computer-Aided Design of Novel Quinazolin-2,4-dione Derivatives as Potential Inhibitors Against *Vibrio cholerae* Click here for additional data file. Supplemental material, Supplementary__File for Synthesis, Characterization, Antibacterial Activity, and Computer-Aided Design of Novel Quinazolin-2,4-dione Derivatives as Potential Inhibitors Against *Vibrio cholerae* by Mohamed El-Naggar, Mahmoud Eldeeb Mohamed, Ahmed Mohamed Mosallam, Wesam Salem, Huda RM Rashdan and Aboubakr Haredi Abdelmonsef in Evolutionary Bioinformatics
