# Supplementary material for: Synthesis, Characterization, Antibacterial Activity, and Computer-Aided Design of Novel Quinazolin-2,4-dione Derivatives as Potential Inhibitors Against Vibrio cholerae
Source: Evol Bioinform Online. 2020 Jan 6;16:1176934319897596. doi: 10.1177/1176934319897596 (PMC6945456; doi:10.1177/1176934319897596)
Supplement: Supplementary__File – Supplemental material for Synthesis, Characterization, Antibacterial Activity, and Computer-Aided Design of Novel Quinazolin-2,4-dione Derivatives as Potential Inhibitors Against Vibrio cholerae [file Supplementary__File.pdf]

Supplementary information

Table of Contents

**List of Contents**	**Page no.**
Figure S1. ^{1}H -NMR of compound 1	2
Figure S2. ^{13}C-NMR of compound 1	2
Figure S3. MS of compound 1	2
Figure S4. ^{1}H-NMR of compound 2	3
Figure S5. ^{13}C-NMR of compound 2	3
Figure S6. MS of compound 2	3
Figure S7. ^{1}H-NMR of compound 3	4
Figure S8. ^{1}H-NMR of compound 4	4
Figure S9. IR of compound 5	4
Figure S10. ^{1}H-NMR of compound 5	5
Figure S11. ^{1}H -NMR of compound 6	5
Figure S12. ^{13}C of compound 6	6
Figure S13. ^{1}H of compound 7	6
Figure S14. ^{13}C of compound 7	6
Figure S15. ^{1}H of compound 8	**7**
Figure S16. ^{13}C of compound 8	**7**
Figure S17. MS of compound 8	7
Figure S18. Minimum inhibitory concentration (MIC) method	8-9

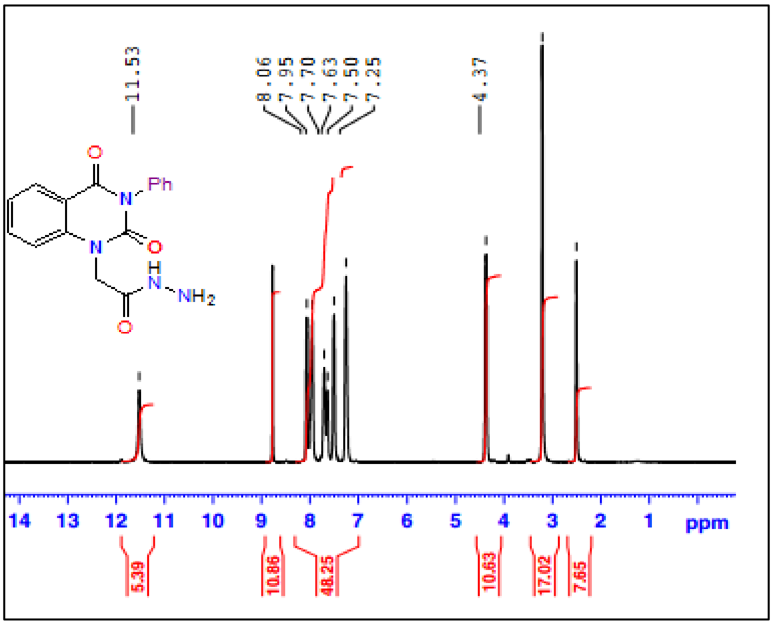

Figure S1. ^{1}H-NMR of compound 1

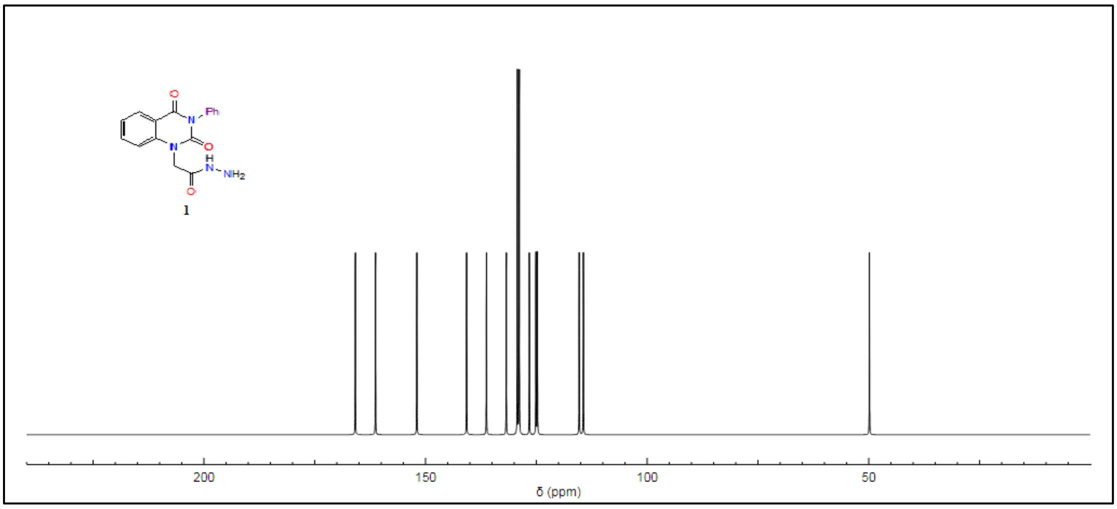

Figure S2. ^{13}C-NMR of compound 1

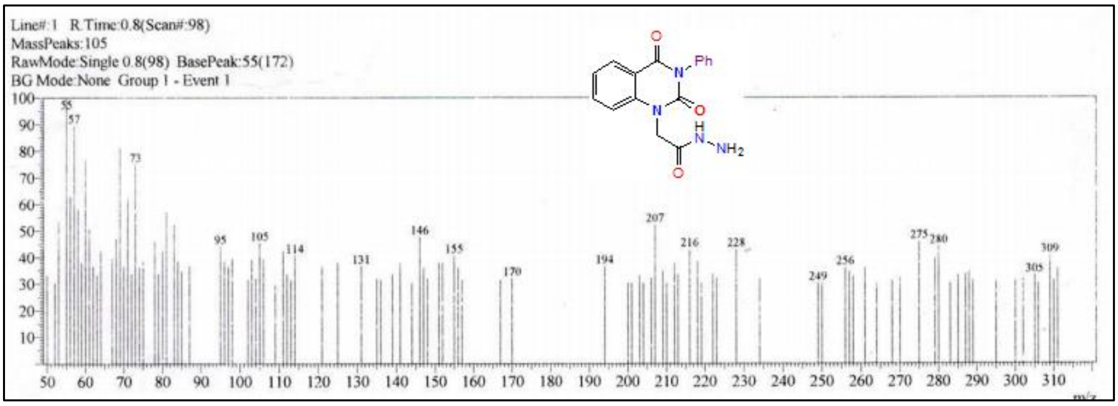

Figure S3. MS of compound 1

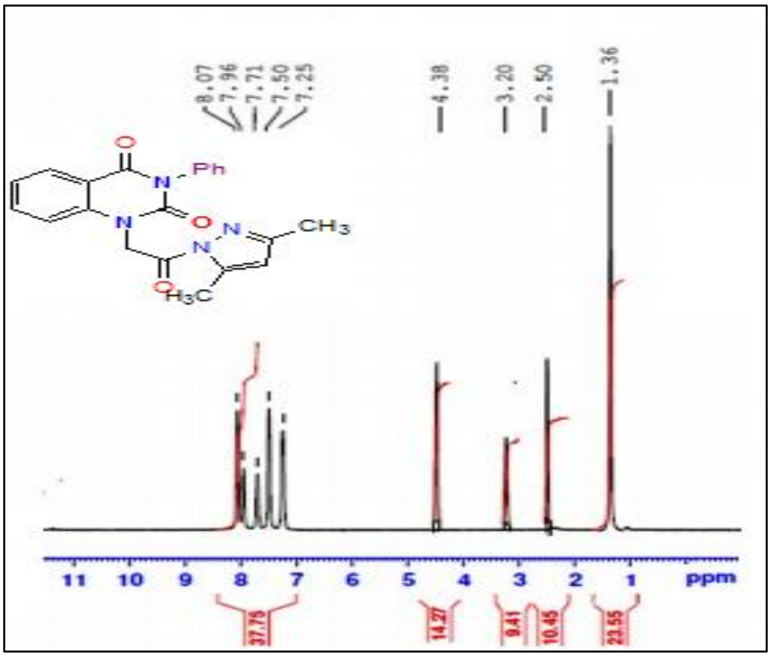

Figure S4. ^{1}H -NMR of compound 2

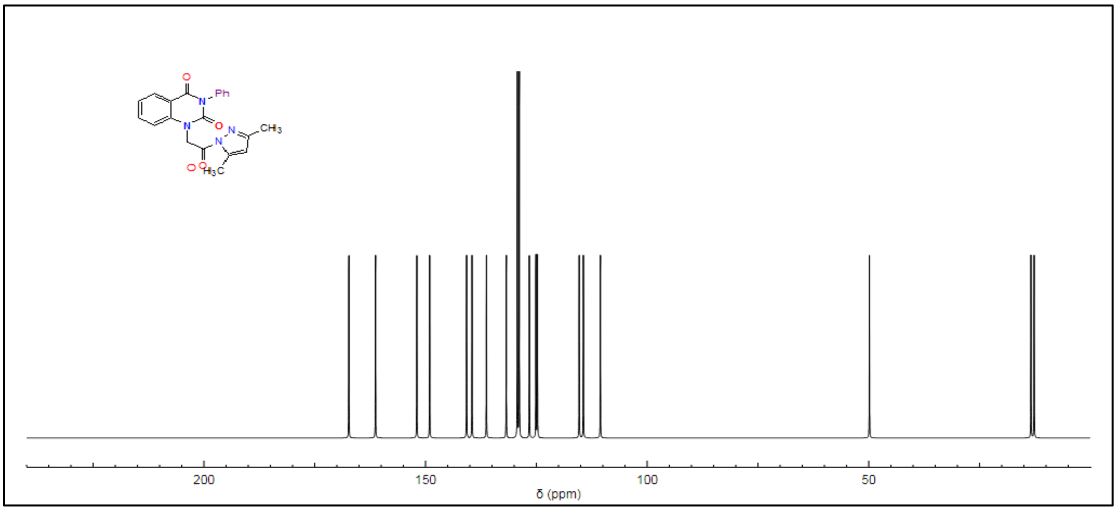

Figure S5. ^{13}C -NMR of compound 2

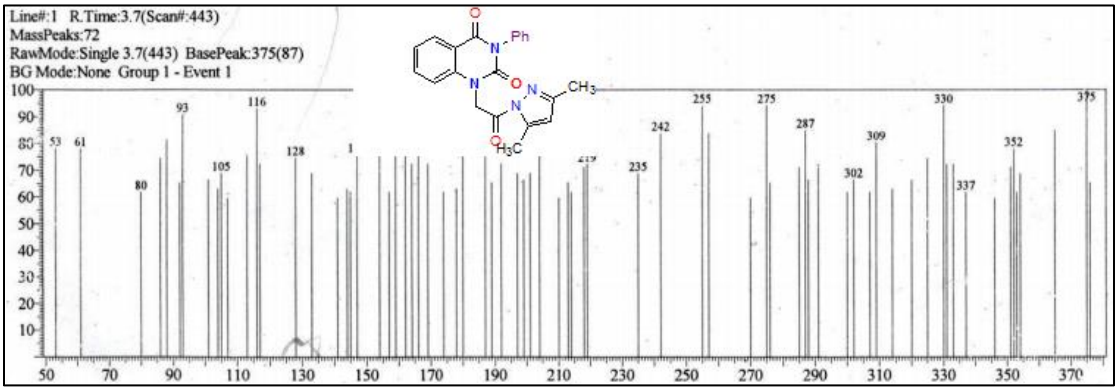

Figure S6. MS of compound 2

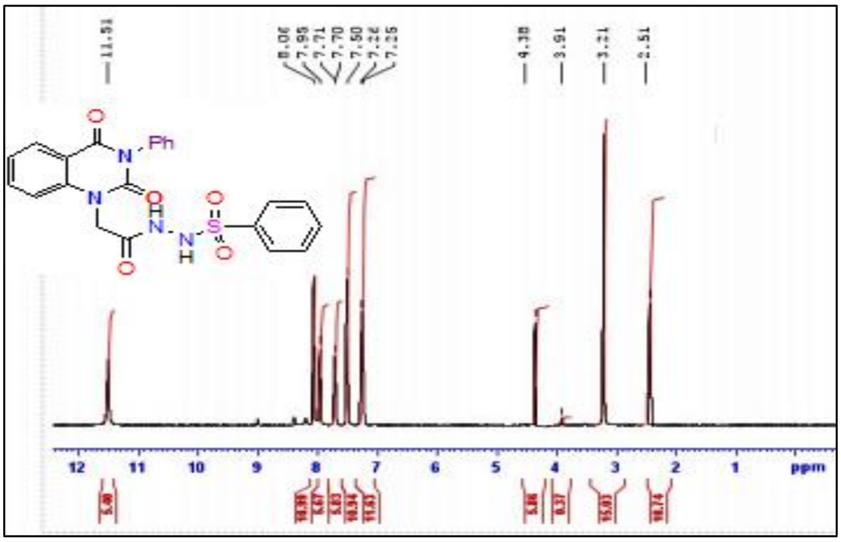

Figure S7. ^{1}H -NMR of compound 3

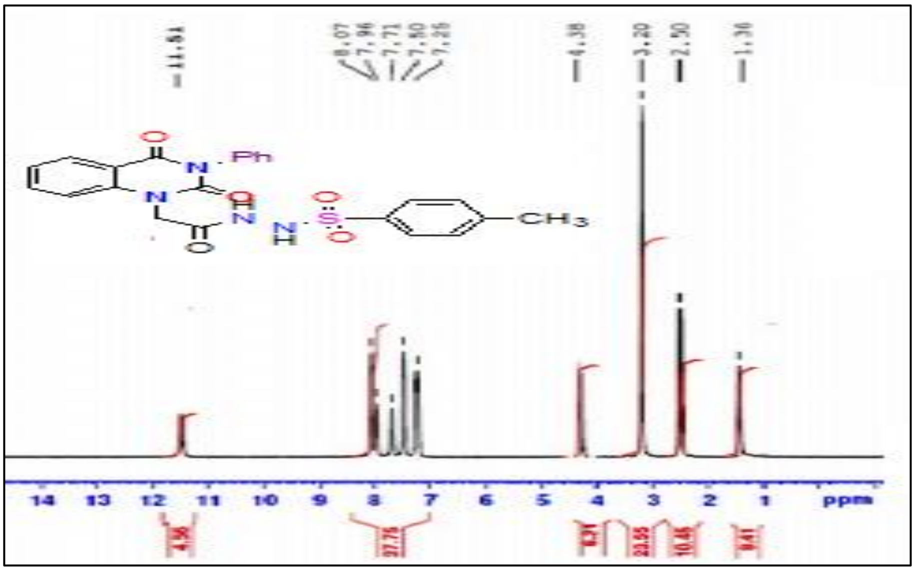

Figure S8. ^{1}H -NMR of compound 4

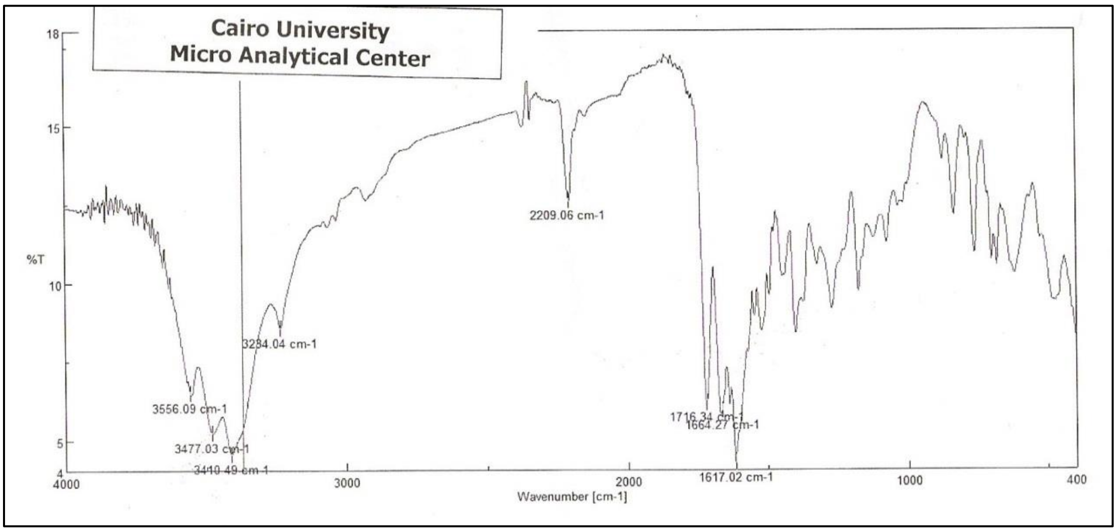

Figure S9. IR of compound 5

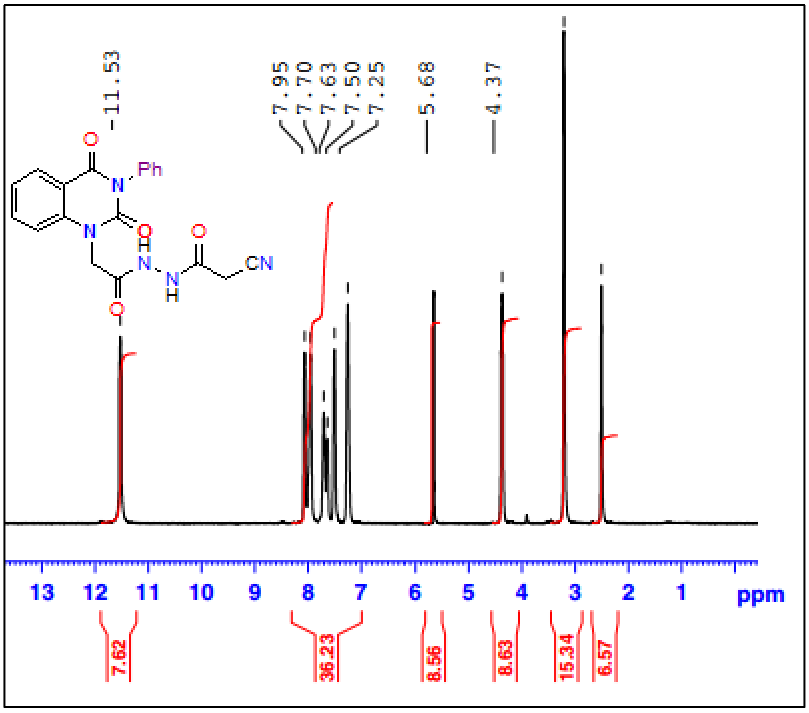

Figure S10. ^{1}H -NMR of compound 5

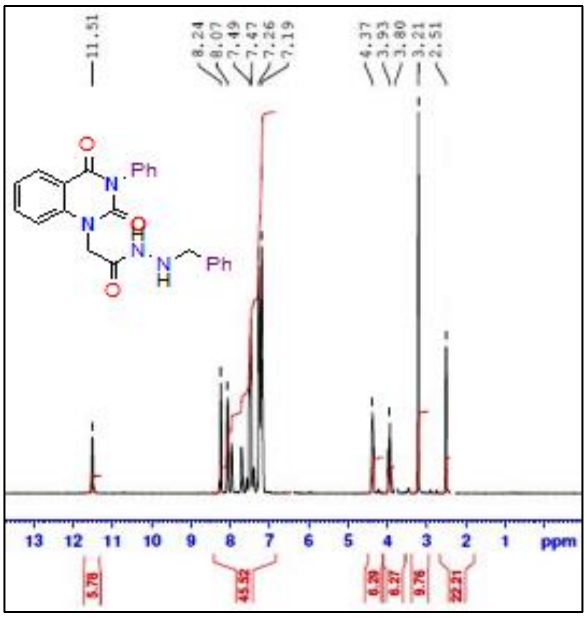

Figure S11. ^{1}H -NMR of compound 6

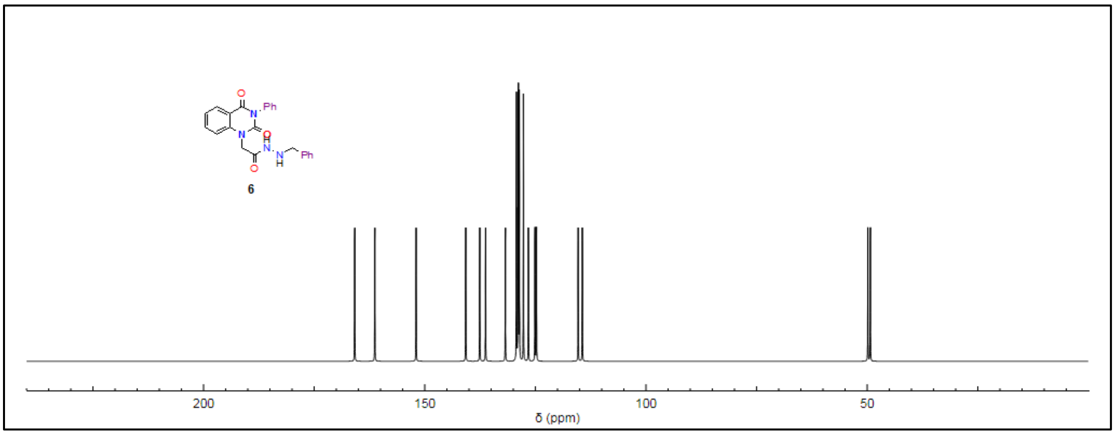

Figure S12. ^{13}C -NMR of compound 6

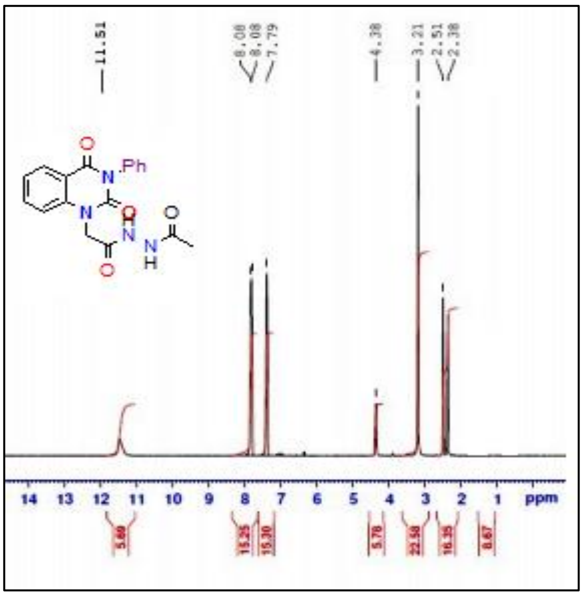

Figure S13. ^{1}H -NMR of compound 7

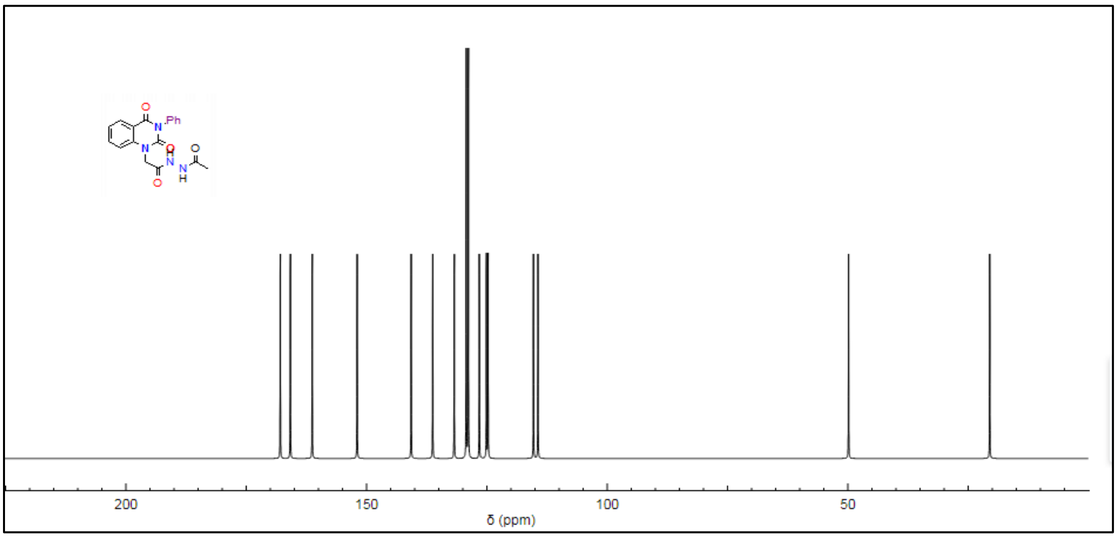

Figure S14. ^{13}C -NMR of compound 7

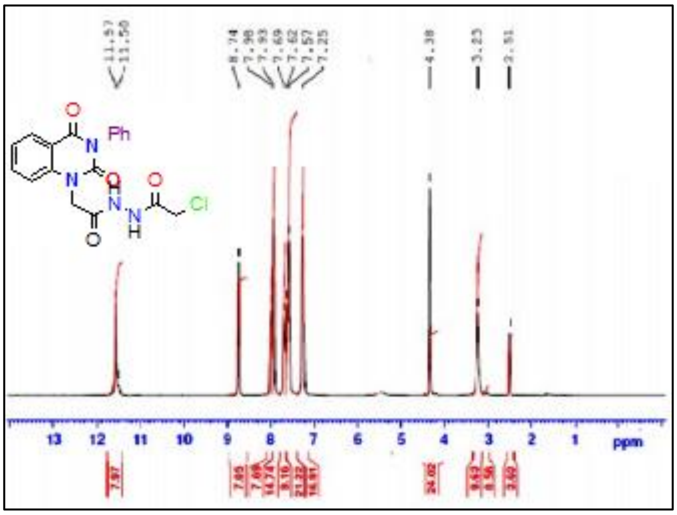

Figure S15. ^{1}H -NMR of compound 8

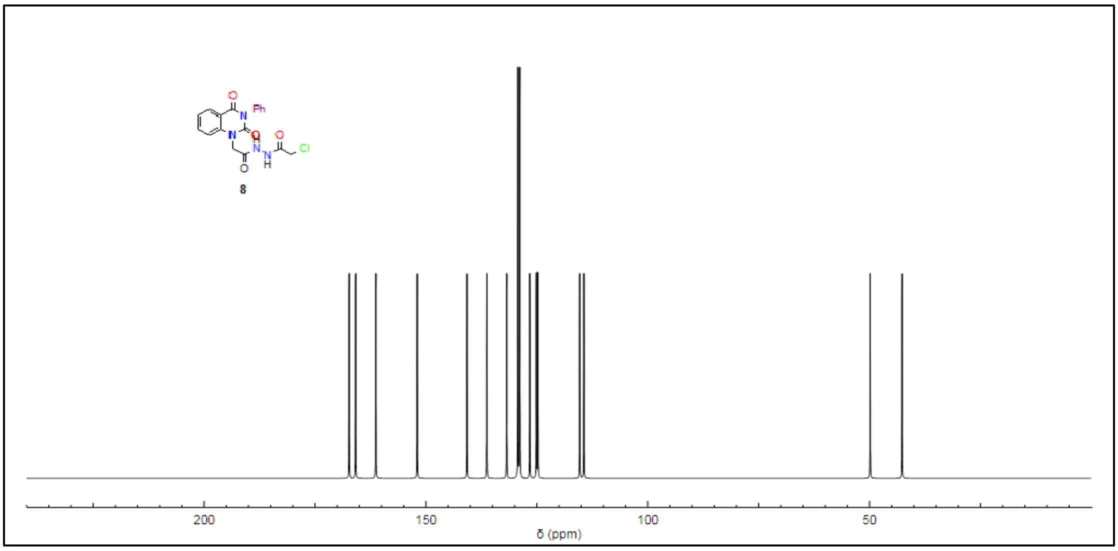

Figure S16. ^{13}C -NMR of compound 8

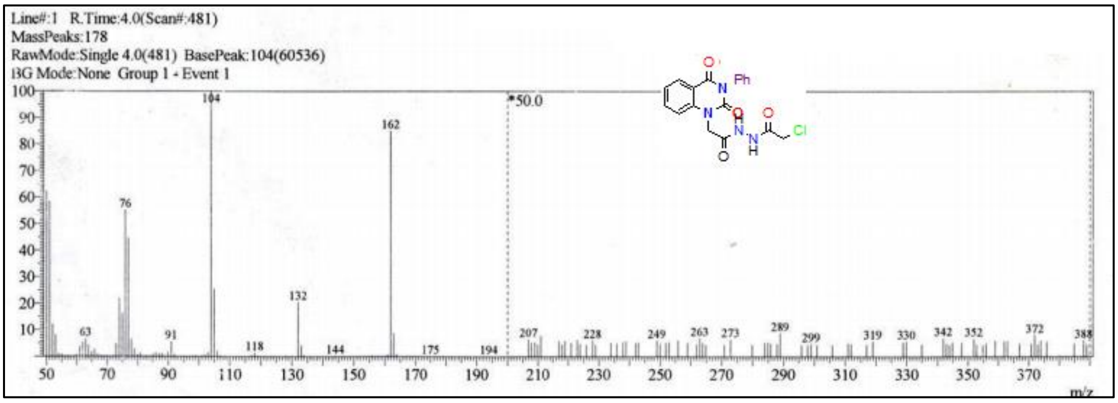

Figure S17. MS of compound 8

Compound 1

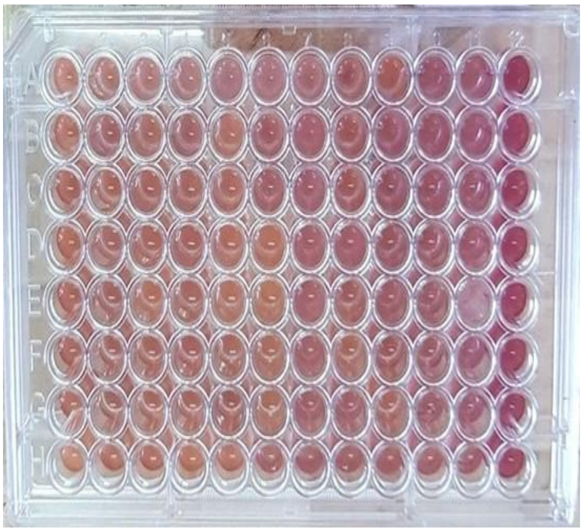

Compound 2

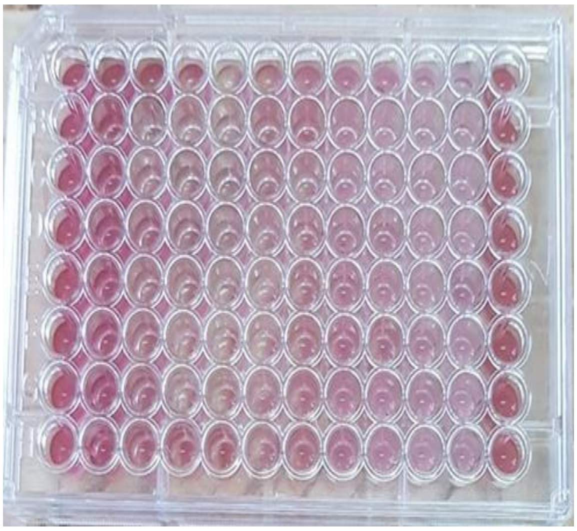

Compound 3

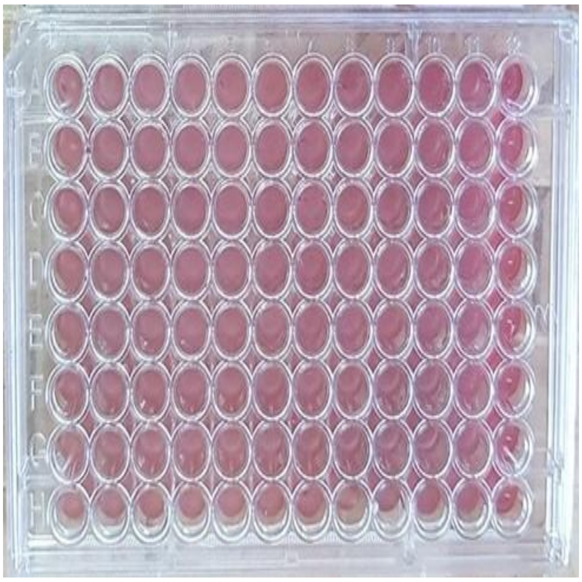

Compound 4

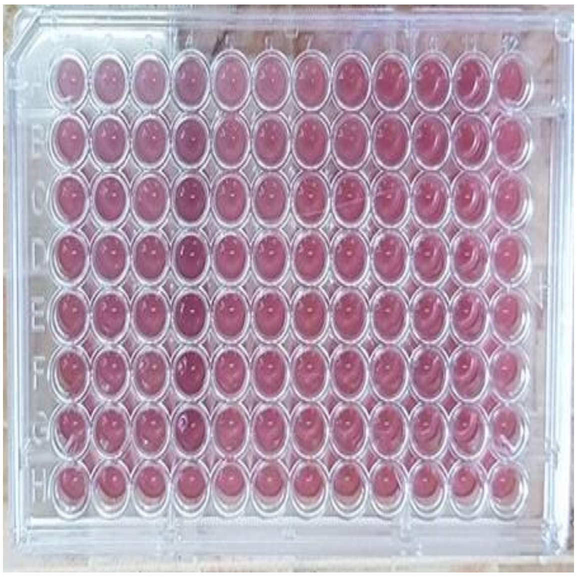

Compound 5

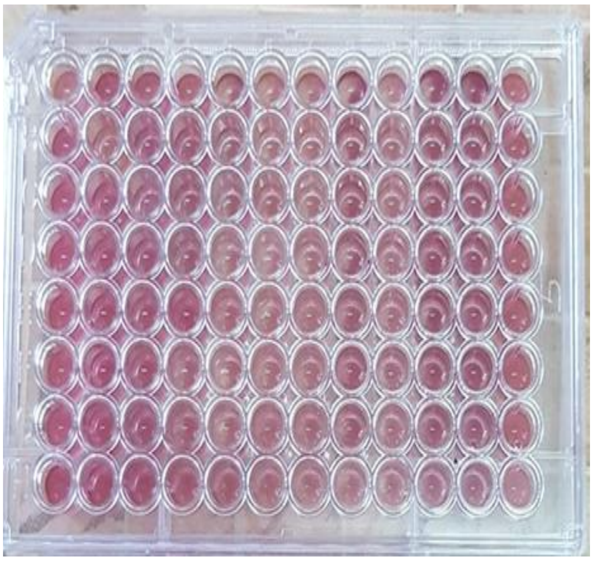

Compound 6

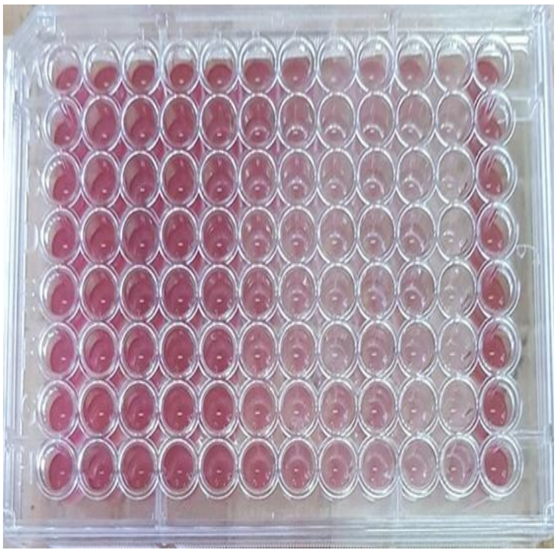

Compound 7

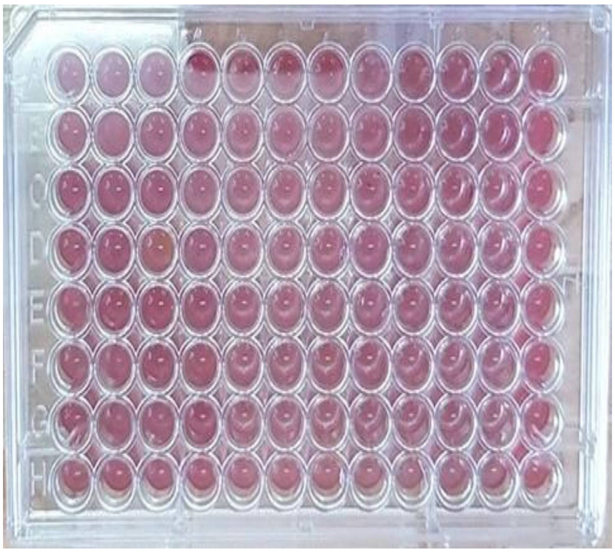

Compound 8

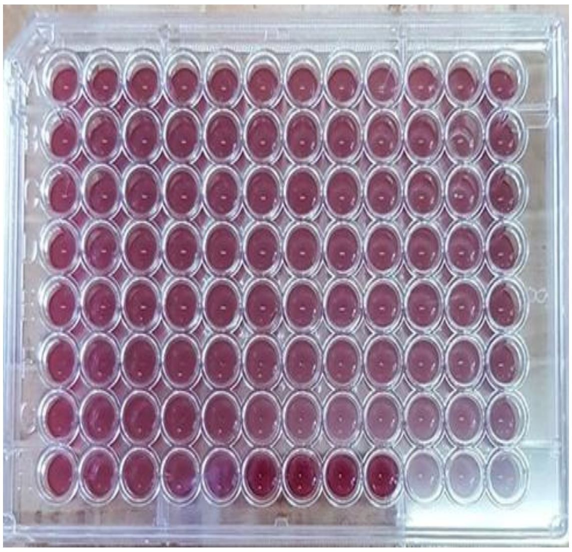

Figure S18. Minimum inhibitory concentration (MIC) method of quinazoline-2,4-dione derivatives **1-8** against Gram-negative *Escherichia coli* O78 strain.
